# Effectiveness and Factors Influencing Success of Transcanalicular Laser-Assisted Endoscopic Dacryocystorhinostomy: Cohort Study

**DOI:** 10.3390/diagnostics14171944

**Published:** 2024-09-03

**Authors:** Radosław Różycki, Łukasz Skrzypiec, Katarzyna Ulaszewska, Jakub S. Gąsior, Jaromir Wasyluk

**Affiliations:** 1 Department of Ophthalmology, Military Institute of Aviation Medicine, 01-755 Warsaw, Poland; radoslawrozycki@gmail.com (R.R.); jarowasyluk@gmail.com (J.W.); 2 Orbita Medical Center, 03-808 Warsaw, Poland; skrzypieclukas@gmail.com; 3 Department of Otolaryngology, Military Institute of Medicine—National Research Institute, 04-349 Warsaw, Poland; 4 Department of Pediatric Cardiology and General Pediatrics, Medical University of Warsaw, 02-091 Warsaw, Poland; gasiorjakub@gmail.com

**Keywords:** lacrimal duct obstruction, LDCR, anatomical success, Munk score, efficacy, lacrimal surgery

## Abstract

Laser dacryocystorhinostomy (LDCR) is a surgical procedure designed to treat obstructions in the lacrimal duct system, which can cause excessive tearing, infections, and discomfort. This technique involves creating a new passage for tear drainage, thereby restoring normal tear flow and alleviating symptoms associated with duct obstruction. A retrospective study was conducted on 48 patients who underwent the LDCR procedure, amounting to 56 eyes. The primary outcome measured was the anatomical success rate, defined as the restoration of duct patency. Patients were examined postoperatively from 6 months to 3.5 years. The LDCR method demonstrated a 95% success rate, encompassing both anatomical and functional outcomes. The procedure’s effectiveness was determined by achieving a patent osteotomy and resolving symptoms. Anatomical success was measured by the creation of a viable drainage pathway, while functional success pertained to the resolution of symptoms such as epiphora. The efficacy of the procedure was found to be independent of both age and gender. Among patients with successful anatomical outcomes, there was a statistically significant improvement in their Munk scores. The LDCR method is highly effective in treating lacrimal duct obstruction. These findings highlight the importance of the Munk score as a predictive indicator of procedural success in LDCR.

## 1. Introduction

Although excessive tearing does not lead to blindness, it is a very bothersome condition. Excessive tearing can have various causes, including reflex tearing due to dry eye, inflammation, allergies, or other ocular surface disorders, as well as reduced tear outflow caused by eyelid malposition, tear pump dysfunction due to eyelid laxity, or obstruction of the nasolacrimal duct [1,2]. It is estimated that 3% of ophthalmology patients visit the emergency room, and a small percentage of them suffer from pathological tearing [3,4]. Of this number, only about 30% of patients will require surgical intervention [5]. Excessive pathological tearing is a frequent disease. Patients often complain that many months or even years pass from the onset of their first symptoms to receiving an accurate diagnosis [6]. During this time, unnecessary antibiotic and anti-inflammatory treatments are often used, leading to unnecessary financial losses and exposing patients to avoidable side effects [7]. The incidence of excessive tearing is estimated to be 1–2 per 100,000 people, meaning that, in Poland, only 200–400 patients will require surgical intervention annually.

Lacrimal duct obstruction refers to a condition in which the natural flow of tears is impeded or obstructed. It is becoming a more common complaint among patients seeking ophthalmologic care, and its associated symptoms can be quite discomforting [8]. The anatomy of the lacrimal drainage system involves several key structures responsible for producing, transporting, and draining tears from the eye into the nose. The process begins in the lacrimal gland, which consists of two lobes: the orbital lobe and the palpebral lobe. Interlobular ducts connect these two lobes. Tear fluid is secreted into the upper conjunctival fornix from both lobes via excretory ducts, and between two and five ducts from the palpebral lobe release fluid into the lower conjunctival fornix [9].

Once tear fluid covers the eye’s surface, it drains into the nasolacrimal duct system. Initially, tears enter through small openings called lacrimal puncta, located at the inner edges of the upper and lower eyelids. From the puncta, tears flow through the lacrimal canaliculi into the lacrimal sac. Tears then travel through the nasolacrimal duct and ultimately drain into the nasal cavity, passing through the valve of Hasner [10].

This intricate system efficiently drains tears, which play a crucial role in protecting and moisturizing the eye’s surface while also removing debris [11]. Stenosis, obstruction, or dysfunction of the lacrimal pump in any part of the system can lead to persistent epiphora. Tear retention may also result from eye or adnexal infections [12]. Persistent tearing, especially in windy weather or with changes in temperature, without signs of lacrimal sac inflammation, may result from reflex tearing due to dry eye syndrome. Additionally, in diagnostics, it is essential to examine for an irregular corneal surface, abnormal eyelid positioning, blepharitis, ocular allergies, and punctal stenosis [13].

Conservative treatment methods do not always yield the desired results, leading to the necessity for surgical intervention. There are various surgical methods available, and the choice of the appropriate technique remains controversial due to the generally good outcomes of each procedure. The differences between techniques are not significant, and each method can substantially benefit the patient [14]. These methods include external dacryocystorhinostomy (External DCR) [15], endonasal dacryocystorhinostomy (EN-DCR) [16], laser dacryocystorhinostomy (LDCR) [17], transcanalicular laser dacryocystorhinostomy (TCL-DCR) [18], endonasal laser dacryocystorhinostomy (ENL-DCR) [19], and conjunctivodacryocystorhinostomy (CDCR) [20].

Surgical success can be described as anatomical and functional, with LDCR, which is the focus of our interest, estimated to yield results of 83.3% and 68.8%, respectively [21]. The main advantages of LDCR surgery include its high success rates, the short duration of the operation, and the absence of visible skin scars. However, the small size of the surgical opening has been considered to be its main drawback, as it may be more prone to earlier reobstruction [22].

The present study aims to present the surgical method and assess the effectiveness of the LDCR procedure in a group of patients operated on at the same clinic by the same operators. We present effectiveness in terms of both surgical and clinical outcomes, as well as an improvement in the Munk Score.

## 2. Materials and Methods

### 2.1. Study Design

This study presents the results of follow-up examinations conducted on patients who underwent LDCR surgery at the same center. Patients included in the study were those who underwent LDCR surgery at the clinic within the past 6 months to 3.5 years. Patients were excluded from the study if they were unable to attend the clinic in person for the examination. Out of 156 patients from the clinic’s database initially eligible for the study, 48 patients aged between 29 and 81 years expressed willingness to participate and consented to the use of their results for scientific purposes. There were 56 eyes that had been operated on: 8 patients underwent both side surgery procedures simultaneously, while 22 underwent only left- and 18 underwent only right-sided procedures. The surgeries were conducted between May 2021 and April 2023 at the “Orbita” Medical Center in Warsaw, Poland by the same operators—ophthalmologist Radosław Różycki and otolaryngologist Łukasz Skrzypiec. Patients were contacted by phone to invite them for follow-up examinations.

On the day of the follow-up appointment (September/November 2023), each patient was at least six months post-operation. During the visit: (1) consent for the examination was obtained, (2) a subjective assessment of surgical outcomes was conducted, including the number of daily tear wipes with the Munk score [23], (3) patients were asked to complete a satisfaction survey according to the Glasgow Benefit Inventory, and subsequently, (4) patients were invited to the examination room, where canalicular tests, reflux tests, endoscopic assessment of fistula presence, size, and location, as well as a fluorescein disappearance test, were performed. Table 1 presents the Munk scores with which the patients were assessed. During the visit, the patients were asked how often they needed to wipe away their tears, and based on their responses, a corresponding score was assigned.

From the patients’ medical history (5), preoperative information was gathered, including the number of past surgeries, surgery date at the clinic, duration of symptoms in years or months, Munk score, subjective assessment of symptom severity, and preoperative diagnosis. All these pieces of information served as the basis for the calculations used in this study.

According to the Act of 5 December 1996 on the Professions of Physicians and Dentists, no ethical consent was required for this study. The study adhered to the principles of the Helsinki Declaration (1964). Participants did not receive any financial or material benefits for their participation. Information regarding anonymity and the use of data for scientific purposes was specified in the consent forms signed by the participants.

The Shapiro–Wilk test was used to assess the normality of the data distribution.

To assess the correlation between the size of the fistula and age, Spearman’s rank correlation coefficient was calculated. To verify sex differences in the size of the fistula, a Mann–Whitney U-test was used. A Wilcoxon signed rank test was used to evaluate changes in the Munk score after the treatment of patients with and without successful anatomical outcomes. The threshold probability of *p* < 0.05 was used as the level of significance for all statistical tests. Statistical analyses were performed using STATISTICA 13 (StatSoft Inc., Tulsa, OK, USA).

### 2.2. Surgical Technique

Transcanalicular dacryocystorhinostomy is performed under intravenous sedation. Additionally, the conjunctival sac is locally anesthetized using 0.5% proxymetacaine hydrochloride. The mucous membrane of the nasal cavity at the site of fistula creation is infiltrated with 2% lidocaine with adrenaline at a ratio of 1:100,000. Next, the lacrimal punctum is dilated with a Nettleship dilator. The lacrimal canaliculus is probed with a Bowman probe and the lacrimal passages are irrigated, removing any pathological secretions from the lacrimal ducts.

After preparing the 400-micrometer diameter optical fiber by removing the sheath from its tip, it is inserted into the lacrimal canaliculus. Upon laser activation, the fiber emits strong red light, allowing for the precise positioning of the working tip within the lacrimal passages. The thin optical fiber is flexible, ensuring good maneuverability during fistula creation. Once the optical fiber is inserted into the lacrimal canaliculus and sac, the working tip is rested against the lacrimal bone. The ENT physician visualizes the site from the nasal cavity using a 45-degree endoscope to determine the correct placement for the laser energy application. If the red marker is in the correct position, the procedure is initiated.

Fistulas are created by tissue evaporation at the site of laser energy application. A Diomed 15 Plus Diode Surgical laser Angiodynamics 810 nm ELVT Vascular 2005 (Diomed Inc., Cambridge, UK) is used for this purpose. Based on the literature and exercises using thermography, a protocol has been developed that establishes the average laser power at 10 watts [18]. Subsequently, the lacrimal sac fistula is created by maneuvering the optical fiber to achieve an approximately 6 mm × 8 mm opening in the lacrimal sac. The ENT physician secures the nasal mucosal tissues with a metal sucker to prevent burns. After opening the lacrimal sac, the lacrimal passages are irrigated, and any pathological secretions are thoroughly aspirated to prevent surrounding tissue infection. The average energy application time is approximately 60–100 s. Probing of the lacrimal passages at the end of the procedure helps to confirm the correct fistula size. The lacrimal passages are intubated with silicone Crawford probes (FCI a Zeiss company, Paris, France) inserted through the upper and lower canaliculi and tied off in the nasal cavity. The procedure concludes with irrigation of the fistula with methylprednisolone acetate at 40 mg/mL.

During the postoperative period, the patient applies antibiotic and steroid eye drops three times daily for three weeks to the operated eye conjunctival sac. Nasal cavity hygiene on the operated side includes saline irrigation four times daily. Generally, clindamycin at 300 mg three times daily for five days is prescribed as an antibiotic. Paracetamol at 500 mg is prescribed for pain relief twice daily for three days post-surgery. According to follow-up examinations, analgesics are rarely needed by patients. Routine postoperative visits are not planned; those that occur are for patients reporting adverse effects. The removal of the Crawford probes is scheduled for three months post-operation. On this day, the fistula quality and patency are also assessed via endoscopic examination according to a predetermined protocol.

### 2.3. Patients Characteristics

Our results present a study involving 48 patients, including 33 females (69%) and 15 males (31%). The average age of the participants was 65 at the time of surgery (ranging from 29 to 81). The median duration of symptoms was 3 years (ranging between 6 months and 50 years). A total of 56 eyes were operated on, with 28 on the right side and 28 on the left side. According to the literature, indications for surgery include the following conditions: primary acquired nasolacrimal duct obstruction, secondary acquired nasolacrimal duct obstruction, persistent congenital nasolacrimal duct obstruction, functional nasolacrimal duct obstruction, acute dacryocystitis unresponsive to medical treatment, and chronic dacryocystitis [24]. For 5 of the operated eyes, this was a second surgery; the remaining eyes were being operated on for the first time. The average Munk symptom score was 4.66, indicating that the patients wiped their tears from 10 times a day or suffered constant tearing. The burden of tearing, rated on a scale from 1 to 10, was, on average, 9. Regarding eyelid evaluation, anatomically correct positioning was observed in 50 patients, while punctal eversion was present in 6 patients.

The surgeries were conducted between May 2021 and April 2023. The postoperative study described in this work was carried out at least 6 months after the surgeries, specifically, in September 2023 and November 2023. For the patients, this ranged from 6 months to 3.5 years post-surgery.

The characteristics of the patients are presented in Table 2.

## 3. Results

### 3.1. Effectiveness of LDCR Procedure

The LDCR procedure demonstrated a 95% success rate in achieving anatomical treatment effectiveness. Anatomical success was defined as the presence of a patent fistula in postoperative examinations. Specifically, success was achieved in 53 eyes, but was not obtained in 3 eyes (5%). The effectiveness of the procedure (measured by the size of the fistula) was found not to be correlated with age (R = 0.071, *p* = 0.611) and not related to the sex of the patients (*p* = 0.317). The classification of the patients’ lacrimal canaliculi characteristics, divided by surgical success and failure, is presented in Table 3.

In the group of patients who achieved successful anatomical outcomes, functional success was also attained. This success was not observed in the group with persistent obstruction. Evidence of functional success is supported by a statistically significant (*p* < 0.001) difference in the Munk scores before (median: 5 points) and after surgery (median: 0 points) among the patients who achieved anatomical success. Specifically, patients with patent fistulas showed marked postoperative improvements in their Munk scores, indicating a significant reduction in symptoms.

Conversely, among those who did not achieve anatomical success, there was no statistically significant difference (*p* = 0.109) in their Munk scores after the procedure (median before: 3 points vs. median after: 1 point). This lack of improvement underscores the continued functional impairment in this group, highlighting the critical link between anatomical and functional success in the LDCR procedure.

The reason for the failures among our three patients was the healing of the postoperative site after the removal of the silicone tube. The fistula sites closed up due to granulation tissue formation.

### 3.2. Effectiveness Based on Time since Surgery

In Table 4, we present the postoperative study results, categorizing the patients based on the time elapsed since their surgery. Two of the unsuccessful surgical outcomes were from patients within 6 months post-surgery, while one was from the period between 1 and 2 years post-surgery. Confirmation of the presence of a fistula and, thus, surgical success was indicated by a positive canalicular test result. A result of a completely patent or partially patent fistula indicated success, whereas a partially obstructed or completely obstructed fistula indicated failure. Each patient during their visit 3 months after their surgery to remove their Crawford probes had patent tear ducts.

## 4. Discussion

In this study, anatomical and functional success rates of 95% were achieved and described within a follow-up period ranging from 6 months to 3.5 years post-surgery.

During the surgery, we employed silicone intubation tubes at the site of the created fistula for all patients. Despite the long-standing nature of this procedure, researchers are still striving to determine whether the use of implants provides superior outcomes compared to their absence [25]. A study comparing patients who underwent endonasal DCR with and without silicone intubation demonstrated a significant benefit for the group that received silicone intubation [26].

It has been reported that, as early as the 18th century, a surgery resembling the modern DCR method was performed in England. It involved drilling a hole in the lacrimal bone and leaving a metal drain in it. It is estimated to have been effective in 70–85% of cases [27].

In 1990, the first case of using the LDCR method in a patient with nasolacrimal duct obstruction was described. Over a 10-month observation period, several advantages compared to traditional methods were noted, including minimal tissue damage and a high procedure efficacy [28]. Laser surgery, compared to other methods, is characterized by a shorter operation time (27 ± 5.5 min). Among complications, lower canaliculus damage and skin defects in the medial canthal region have been noted [29]. In most retrospective studies, anatomical success is achieved in 70% to 90% of cases.

There are retrospective studies that have also evaluated the long-term effectiveness of LDCR treatment. At a tertiary referral hospital, with 92 lacrimal ducts included, the one-year anatomical and functional success rates were 71% and 65.5%, respectively, depending on the modification applied during the surgery. The intraoperative event rate was 3.63%, and the postoperative complication rate was 3.27% [30]. In a subsequent study, success was defined as the complete resolution of epiphora and the observation of fluid flow without any anatomical obstruction during lacrimal system irrigation. Among patients who underwent the laser procedure, a success rate of 78.5% was achieved. The patients in both groups were monitored for at least 4 years, and a high efficacy of the surgery was still observed [31]. In a follow-up study of 40 patients aged from 21 to 85 years, evaluated after 72 months, 27 of the 40 patients (67.5%) were female and 13 (32.5%) were male. Nasolacrimal duct obstruction (NDO) was right-sided in 17 patients (42.5%), left-sided in 22 patients (55%), and 1 patient (2.5%) underwent bilateral surgery. After 5 years, anatomical success was observed in 75% of the patients, while functional success was noted in 65% [32]. The patients were evaluated at 3 months, 6 months, 1 year, 3 years, and 5 years postoperatively. A gradual decrease in effectiveness was observed, starting at 90% at 3 months after surgery. In our study, the effectiveness was 100% at 3 months for all patients, but during follow-up, it was 81.82% for those evaluated at 6 months, 100% for those evaluated between 6 months and 1 year, 95.45% for those evaluated between 1 and 2 years, and 100% for those evaluated between 2 and 3.5 years. Unfortunately, a precise comparison of results is not possible, because our follow-up did not involve the same patients at different time intervals. In another study, a complete resolution of symptoms was observed in 85.4% of cases after 3 months. Functional success was defined as the resolution of epiphora and the presence of a patent ostium during lacrimal irrigation. Anatomical success was defined as a patent ostium with continued epiphora. The functional success rate decreased to 67.7% at 6 months, 63.3% at 1 year, and 60.3% at 2 years, while the patency of the lacrimal drainage system was restored in 93.1%, 74.6%, 69.5%, and 68.2% of cases, respectively. Consistent with our study, no correlation was found between patient age and effectiveness. Additionally, no correlation was found between effectiveness and the amount of laser energy used [33]. In our study, each patient achieved patency of the tear ducts after three months, likely due to the use of intubation at the site of the fistula. For comparison, in a study that compared external and endoscopic DCR procedures, which were performed on 618 and 185 patients, respectively, the success rates were 92.4% and 91.1% [34]. In a study involving 165 patients and 213 eyes, a significant variation in the duration of symptoms before surgery was also observed, ranging from 1 month to 50 years. It was not demonstrated that the duration of symptoms affected the surgical outcome—similar to our findings. An effectiveness rate of 85% was achieved with the DCR method [35].

In a study of 118 LDCR procedures, 27 were deemed unsuccessful due to the recurrence of epiphora, discharge, or reflux during nasolacrimal irrigation. One of these failures was permanently resolved with balloon dacryoplasty. Nine other failures underwent repeat procedures, with seven remaining patents after one repeat procedure, and an additional one remaining patent after a third procedure. Among the initial 102 lacrimal surgeries in this series, the success rate was 73.6%. The overall success rate, including repeat procedures, was 81.5% [36]. In an analysis of intraoperative and postoperative complications, issues such as the inability to position the laser instrument during surgery, thermal injury to the canaliculus (rare), canalicular infection, or silicone tube prolapse (most common) were described. A significant number of patients who did not achieve anatomical success required a second surgery [37]. The LDCR procedure is also characterized by a lower risk of infection and bleeding compared to other methods [38]. The greatest advantage of the LDCR procedure, as reported, is its short duration and the patients’ good conditions post-operation, allowing them to be discharged on the same day [39]. In our study, a higher average effectiveness was achieved. This could be attributed to the consistent composition of the surgical team, which may reduce the likelihood of failure. Additionally, patients receiving private healthcare are often more cooperative and compliant with recommendations [40]. The high effectiveness and low rate of failure may also be attributed to adherence to guidelines, including the use of mitomycin, selective steroid therapy, and hygiene in the days following the surgery.

Among pediatric patients, 18 children who underwent LDCR surgery on 20 lacrimal ducts were considered. All patients had a history of epiphora and chronic dacryocystitis; two patients (10%) had acute dacryocystitis. Previous treatments included probing and irrigation in all eyes (100%) and silicone tube intubation in nine eyes (45%). The patients were retrospectively evaluated 14–24 months after the procedure, achieving a 100% anatomical success rate and an 85% clinical success rate. In our practice, the success rates were 95% anatomical and 95% clinical [41].

It is interesting to note that postoperative success rates decline with the passage of time after surgery. Additionally, the further the follow-up date is from the surgery, the fewer patients are willing to return for evaluation, making it challenging to perfectly assess the outcomes. In a study involving 205 patients, it was found that, similar to our findings, functional success is not dependent on gender, age, laser energy, or the duration of the surgery. However, the pain scale experienced during the operation is influenced by both the laser energy and the duration of the surgery [42]. Despite its advantages, laser-assisted DCR has limitations. Excessive laser energy can cause scarring and secondary obstruction. The heat used to vaporize bone may lead to granulation tissue formation, potentially causing secondary blockage. Precise guidelines for laser energy, pulse duration, and pauses are still undefined. There is also a risk of thermal injury [37,43].

## 5. Conclusions

This study demonstrates that the LDCR method is a highly effective treatment for lacrimal duct obstruction. For patients, these findings offer reassurance regarding the effectiveness and reliability of the LDCR procedure, potentially leading to an increased acceptance and preference for this treatment option. The high success rate means that a wider range of patients can benefit from this procedure, experiencing substantial relief from the symptoms of lacrimal duct obstruction and an overall improvement in quality of life.

For clinicians, this study provides valuable insights into the efficacy of LDCR, reinforcing its status as a preferred treatment method. The independence of success from demographic variables simplifies patient selection and preoperative planning, allowing for more straightforward decision-making processes. Additionally, the use of Munk scores as a predictive tool can aid in postoperative assessment and patient counseling, enhancing the overall management strategy for individuals undergoing this procedure.

In the long term, these findings may encourage further research into optimizing LDCR techniques and exploring their applicability in various clinical scenarios. As the understanding of the procedure’s efficacy expands, it could lead to the development of standardized protocols and guidelines, ultimately improving patient outcomes on a broader scale. Thus, the study not only highlights the current benefits of LDCR, but also paves the way for future advancements in the treatment of lacrimal duct obstruction.

## Figures and Tables

**Table 1:** Munk score.

Grade	Patient’s Answer
0	No epiphora.
1	Tearing that causes wiping away of tears less than twice a day.
2	Tearing that causes wiping away of tears 2–4 times a day.
3	Tearing that causes wiping away of tears 5–10 times a day.
4	Tearing that causes wiping away of tears more than 10 times a day.
5	Constant epiphora.

**Table 2:** Patients’ characteristics.

Variable		Patients [*n* = 48]
Age [years]		65
Gender	Female	33 (69%)
	Male	15 (31%)
Operated side	Only right	18 (37.50%)
	Only left	22 (45.83%)
	Right and left	8 (16.67%)
Diagnostic	Chronic dacryocystitis	21 (43.75%)
	Sacculated dilation and nasolacrimal duct obstruction	13 (27.08%)
	Acute dacryocystitis unresponsive to medical treatment	3 (6.25%)
	Functional nasolacrimal duct obstruction	2 (4.17%)
	Nasolacrimal duct obstruction	9 (18.75%)

**Table 3:** Characteristics of the patients’ lacrimal pathways postoperatively.

Variable		Patent Lacrimal Duct [*n* = 53]	Obstructed Lacrimal Duct [*n* = 3]
Mean age		62	64.7
Operated side	Right	26 (49.1%)	2 (66.7%)
	Left	27 (50.9%)	1 (33.3%)
Average Munk score	Before surgery	4.72	3.66
	After surgery	0.38	1.33
Average fistula size in mm		2.03	0.17

**Table 4:** Categorization of patients by time since surgery.

Variable		6 Months Postoperatively [*n* = 11]	6 Months–1 Year Postoperatively [*n* = 18]	1–2 Years Postoperatively [*n* = 22]	2–3, 5 Years Postoperatively [*n* = 5]
Duration of symptoms before surgery	<1 year	1 (9.09%)	4 (22.22%)	0 (0%)	0 (0%)
	1–5 years	5 (45.45%)	10 (55.56%)	11 (50%)	3 (60%)
	> 5 years	5 (45.45%)	4 (22.22%)	11 (50%)	2 (40%)
Postoperative canalicular test:	Completely patent	8 (72.73%)	15 (83.33%)	19 (86.36%)	4 (80%)
	Partially patent	1 (9.09%)	3 (16.67%)	2 (9.09%)	1 (20%)
	Partially obstructed	1 (9.09%)	0 (0%)	0 (0%)	0 (0%)
	Completely obstructed	1 (9.09%)	0 (0%)	1 (4.55%)	0 (0%)

## Data Availability

Data is available during the contact with Orbita Medical Center in Warsaw kontakt@centrummedyczneorbita.pl.
